# Case report: Two pediatric cases of long-term leukemia-free survival with relapsed acute T-lymphoblastic leukemia treated with donor CD7 CAR-T cells bridging to haploidentical stem cell transplantation

**DOI:** 10.3389/fimmu.2024.1333037

**Published:** 2024-02-28

**Authors:** Yanzhi Song, Zhanxiang Liu, Qi Wang, Kong Gao, Tong Wu

**Affiliations:** Department of Bone Marrow Transplantation, Beijing Boren Hospital, Beijing, China

**Keywords:** T-lymphoblastic leukemia, CD7 CAR-T, long-term survival, leukemia-free survival, allogeneic stem cell transplantation

## Abstract

**Introduction:**

Patients with relapsed/refractory (r/r) acute T-lymphoblastic leukemia (T-ALL) have a poor prognosis. We developed donor CD7 chimeric antigen receptor T (CAR-T) cells to salvage r/r T-ALL patients and obtained encouraging results. Patients who had not received allogeneic (allo-) hematopoietic stem cell transplantation (HSCT) before CAR-T therapy would develop pancytopenia and immunodeficiency for a long period after CD7 CAR-T therapy; therefore, allo-HSCT is needed in these patients. Here, we report two pediatric r/r T-ALL patients who received donor CD7 CAR-T bridging to allo-HSCT with leukemia-free survival (LFS) and sustained negative minimal residual disease for >2 years.

**Case presentation:**

Patient 1 was a 10-year-old boy who visited our hospital because of a T-ALL relapse with multiple lymphadenopathies without discomfort. The patient did not achieve remission after one course of induction chemotherapy. The patient then received donor (his father) CD7 CAR-T cells and achieved complete remission (CR). Thirty days after the first CAR-T cell infusion, he received allo-HSCT, and his father was also the donor. His LFS was >3 years. Patient 2 was an 8-year-old boy who was admitted to our hospital with relapsed T-ALL with fever, cough, and mild dyspnea. He did not achieve remission after one course of induction chemotherapy; therefore, he received donor (his father) CD7 CAR-T cells and achieved CR. Twenty-six days after CAR-T cell infusion, the patient received allo-HSCT, with his father as the donor. He has survived for >2 years free of leukemia. At the last follow up, both patients were alive and presented a good quality of life

**Conclusion:**

The long-term survival of these two patients supports the use of CD7 CAR-T therapy bridging to allo–HSCT as an effective and safe treatment with the capacity to make r/r T-ALL a curable disease, similar to r/r acute B-lymphoblastic leukemia.

## 1 Introduction

The treatment of relapsed/refractory (r/r) acute B-lymphoblastic leukemia (B-ALL) has been revolutionized with blinatumomab, inotuzumab, ozogamicin, and chimeric antigen receptor T (CAR-T) cell therapy. As a result of these new methods, many patients with r/r B-ALL can achieve remission (1–4), and when bridged to allogeneic (allo-) hematopoietic stem cell transplantation (HSCT), more than 60% of the patients can achieve long-term survival (5, 6). However, at present, there are no new therapeutic methods that are as effective in r/r acute T-lymphoblastic leukemia (T-ALL). Therefore, most patients with r/r T-ALL do not achieve remission and have dismal outcomes.

Recently, we developed donor-derived CD7 CAR-T cells to salvage r/r T-ALL patients and almost 90% of patients achieved complete remission (CR) after CAR-T therapy (7). However, patients who had not received allo-HSCT developed pancytopenia and immunodeficiency for a long period of time (7); thus, these patients should be prescribed allo-HSCT after CAR-T. Additionally, there was a number of differences in treatment details between r/r T-ALL received donor CD7 CAR-T bridging to allo-HSCT and r/r B-ALL received CAR-T bridging to allo-HSCT. Here we report two cases of pediatric r/r T-ALL patients who underwent donor CD7 CAR-T in the phase I clinical trial (ChiCTR2000034762 and NCT04689659) bridging to allo-HSCT and survived for >2 years without leukemia relapse. To the best of our knowledge, Patient 1 is the first reported T-ALL patient to receive CD7 CAR-T cell treatment worldwide, and has survived for >3 years free of leukemia following transplantation. We decided to present these two cases to support the use of CD7 CAR-T cell therapy bridging to allo-HSCT for r/r T-ALL. Indeed, after a considerable follow up, both patients are in long term remission and present a good quality of life. Moreover, these two cases offer an example of the complex management of the patients treated with this innovative cellular therapy. The case report follows the CARE Checklist (**Supplementary Figure 2**).

## 2 Case presentation and diagnostic assessment

### 2.1 Patient 1

**Figure 1:**
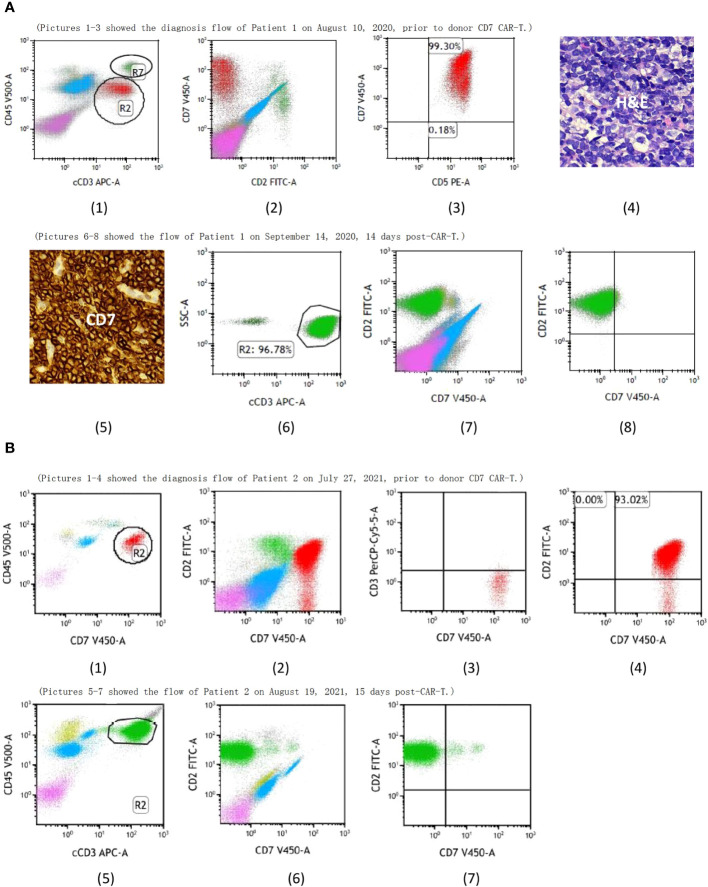
**(A)** (1) R2 showed the T-lymphoblastic leukemia (T-ALL) cells and R7 showed the normal T cells of Patient 1, both of them expressed CD7 prior to donor CD7 CAR-T. Pictures 1-3 showed the bone marrow aspirate evaluated by flow cytometry while pictures 4-5 showed the punctured biopsy pathology of the inguinal lymph node before CAR-T and they demonstrated that the T-ALL cells of Patient 1 expressed CD7. Pictures 6-8 showed CD7+ cells from Patient 1 were undetectable after donor CD7 CAR-T. **(B)** (1) R2 showed the T-ALL cells of Patient 2 prior to donor CD7 CAR-T. Pictures 1-4 showed bone marrow aspirate evaluated by flow cytometry and demonstrated that the T-ALL of Patient 2 expressed CD7. Pictures 5-7 showed CD7+ cells from Patient 2 were undetectable after donor CD7 CAR-T.

In July 2017, a 7-year-old boy was admitted to hospital with paleness and fever; he was subsequently diagnosed with T-ALL. A complete blood cell analysis revealed a white blood cell (WBC) count of 125×10^9^/L. He received two courses of induction chemotherapy to achieve CR. He then received consolidation and maintenance chemotherapy according to the Chinese Children’s Cancer Group Acute Lymphoblastic Leukemia (CCCG-ALL) 2015 protocol (8). He was admitted to our hospital on August 10th, 2020, due to multiple lymphadenopathies without discomfort, bone marrow aspiration was prescribed and revealed 18.5% lymphoblasts expressing CD7 (**Figure 1A** (1–3)) with a normal karyotype. No fusion genes were detected while gene mutations in *NOTCH1* p.F1592delinsLD, *EP300* p.N1752Kfs*131, *KIT* p.M541L, *MED12* p.L1058delinsPQPS, *SH2B3* p.R392Q, or *STXBP2* p.T318M were detected using next-generation sequencing. Puncture biopsy of the inguinal lymph nodes showed T-lymphoblastic cells with myeloid and CD7 expression infiltration (**Figure 1A** (4,5)). He revealed no special personal and family histories. He received induction therapies; however, 17.5% lymphoblasts were observed in the bone marrow smear after chemotherapy.

**Figure 2:**
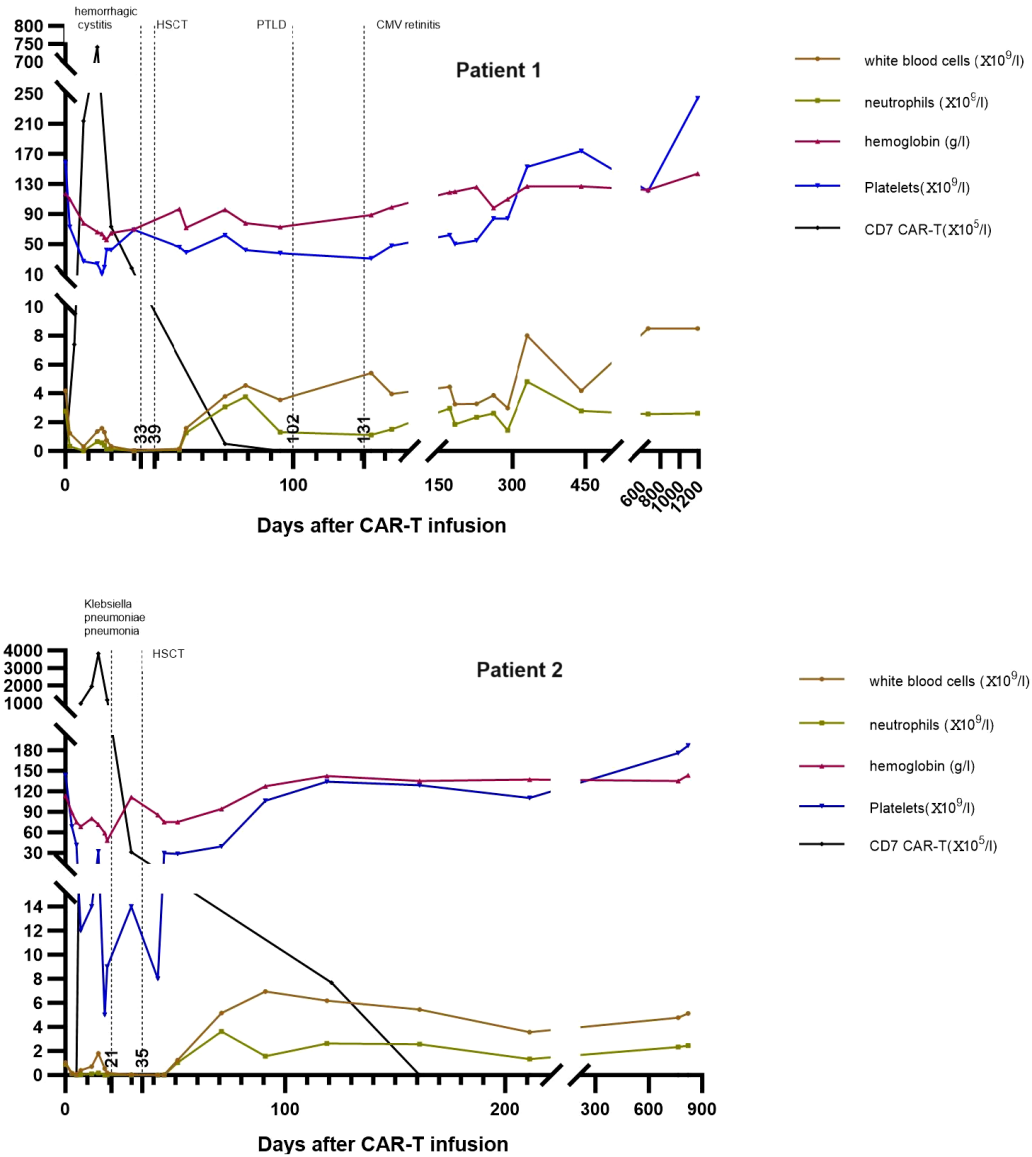
The trends of donor CD7 CAR-T cells and blood cells counts in peripheral blood of Patient 1 and Patient 2. CAR-T, chimeric antigen receptor T; HSCT, hematopoietic stem cell transplantation; PTLD, post-transplant lymphoproliferative disease; CMV, cytomegalovirus.

On August 26th, 2020, a lymphodepletion regimen was administered and then donor-derived (his father) CD7 CAR-T cells were infused. Following CAR-T cell infusion, the patient rapidly developed grade 2 cytokine release syndrome (CRS) (9) which has been reported in Doctor Pan’s report (7). The patient's blood cell count did not recover after CRS; the WBCs increased transiently but decreased to severe granulocytopenia again in less than 10 days (**Figure 2**). Bone marrow aspiration was performed on day 30 post-infusion; and the results showed that the disease was in CR detected by bone marrow smear and multiparametric flow cytometry. The enlarged lymph nodes were evaluated using ultrasonography and enhanced computed tomography (CT). However, pancytopenia did not improve. While CAR-T cells were detected in the peripheral blood, CD7+ T lymphocytes were undetectable (**Supplementary Figure 3**). He withdrew from the trial because of a bridging transplantation on September 30th, 2020. The patient’s father was the HSCT donor. The patient received busulfan, fludarabine, smolastine, and anti-human T-cell lymphocyte rabbit immunoglobulin (ATG, Fresenius Biotech GmbH) (busulfan: 0.8 mg/kg q6h from day -9 to day -7; fludarabine: 30 mg/m^2^/day from day -6 to day -2; smolastine: 250 mg/m^2^ day -3; ATG: total 15 mg/kg from day -5 to day -2) as the transplantation conditioning regimen. Granulocyte colony-stimulating factor (G-CSF)-mobilized donor bone marrow and peripheral blood stem cells (PBSCs) containing mononuclear cells (MNCs) 5.1×10^8^/kg and CD34+ cells (4.4×10^6^/kg) were infused on October 9th and 10th, 2020 (+39 days post-CAR-T). Cyclosporine A (CsA), mycophenolate sodium, and a short course of methotrexate (MTX) were administered to prevent graft-versus-host disease (GVHD). CsA was continuously infused at a dose of 2.5 mg/kg per day from day -9, with adjustments to the infusion speed to maintain a plasma concentration of 100–150 ng/ml in the first month after transplantation, which was then replaced with a low concentration of tacrolimus for 3 months; mycophenolate sodium (6 mg/kg q12h) was used from day -2 to day +28; and MTX was administered at doses of 15 mg/m^2^ on day +1, and 10 mg/m^2^ on days +3, +6, and +11. Neutrophil cells and platelets recovered by day +12 and 3 months post-HSCT, respectively. Complete donor chimerism of both CD3+ T cells and bone marrow nucleated cells was achieved on day +30 post-HSCT, which was analyzed by multiplex PCR using short tandem repeats (STRs).

Following CD7 CAR-T treatment, a GVHD-like reaction was observed 11 days post-infusion, which manifested as a rash on the face and extremities, dark green watery stools, and significantly elevated transaminase and soluble CD25 levels. This condition was controlled with a multidrug combination therapy involving tocilizumab, high doses of methylprednisolone, ruxolitinib, and basiliximab. In addition, reactivation of Herpes virus type 6 (HHV6) occurred and treated with foscarnet sodium before transplantation.

During the HSCT process, hemorrhagic cystitis, which occurred on day 3 after starting conditioning regimen, manifested with grade 3 gross hematuria and blood clots (10, 11), increased urinary frequency, and mild dysuria. Laboratory tests revealed BK virus in the urine, although routine urine and urine culture were normal; therefore, hemorrhagic cystitis caused by viral infection was diagnosed. Despite the intravenous injection of sodium foscarnet and immunoglobulin, hydration, alkalinization, and blood transfusion support, the patient’s condition persisted for 3 months. At +1-month, the patient developed grade II acute GVHD (International Bone Marrow Transplant Registry grading systems) (12), presenting as a rash all over the body and fever that was controlled with glucocorticoids, CsA, and MTX. Twenty days later, Epstein-Barr virus (EBV) was reactivated with fever, ultrasonography revealed multiple superficial lymph node enlargements, and peripheral blood flow cytometry showed 0.9% monoclonal plasma cells. The patient was clinically diagnosed with post-transplant lymphoproliferative disease (PTLD), which was successfully treated with immunosuppressant reduction, immunoglobulin, and two courses of chemotherapy with the R-VP regimen (rituximab 375 mg/m^2^ on day 0, vincristine 4 mg on day 1, and dexamethasone 7 mg on day 1–5). At +3 months, the patient developed blurred vision and photophobia, diagnosed as cytomegalovirus (CMV) retinitis, which improved with intraocular injection of antiviral drugs. At +6 months, the patient developed a headache; magnetic resonance imaging revealed abnormal strengthening foci in the brain, meninges, and temporalis muscles. Furthermore, lumbar puncture showed high cranial pressure, high cerebrospinal fluid protein, and normal leukocytes; no abnormal cells and herpes viruses were found in the cerebrospinal fluid. After mannitol was administered, the headache disappeared, and the imaging lesions disappeared on re-examination and did not reappear. At +20 months, the patient developed mild hypoxemia, and chest CT findings indicated bronchiolitis obliterans with organizing pneumonia and mediastinal emphysema. The patient was diagnosed with chronic GVHD and was treated with methylprednisolone and ruxolitinib. At +24 months, the patient developed joint contracture in both elbow joints combined with elevated bilirubin and was alleviated with ruxolitinib.

After HSCT, we regularly reviewed the bone marrow, peripheral blood, and imaging tests to assess the patient’s condition (+1, +2, +3, +4, +6, +8, +10, +12, +15, +18, +21, +24, +30, and +36 months). The patient has been continuously CR and minimal residual disease (MRD)- detected by flow cytometry for >3 years. The CD7+, CD4+ and CD8+ cells recovery is shown in **Supplementary Figure 3**. CAR-T cells were produced, detected, and quantified as described in our previous report (7).

### 2.2 Patient 2

A 6-year-old boy was diagnosed with T-ALL in May, 2019. He achieved CR after two courses of induction chemotherapy and then received consolidation and maintenance therapies according to the CCCG-ALL 2015 protocol (8). He relapsed in March, 2021; however, he did not achieve remission after induction therapy with a regimen of mitoxantrone, dexamethasone, and vincristine. On July 27, 2021, he visited our hospital with cough, high fever, and mild dyspnea. Chest CT indicated a lung infection, which improved after antibiotic treatment. In addition, bone marrow examination showed 33% T-lymphoblastic cells with CD7 expression (**Figure 1B** (1–4)). Chromosome testing revealed a normal karyotype, with no fusion or mutated genes. Extramedullary diseases were ruled out. He revealed no special personal and family histories.

On July 30th, 2021, lymphodepletion treatment was administered and then donor (his father) derived CD7 CAR-T cells were infused. Following CAR-T cell infusion, the patient rapidly developed grade 2 CRS and infection. which has been reported in Doctor Pan’s report (7). The CRS and infection were controlled with immunosuppresive treatment and powerful broad-spectrum antibiotics. He also developed serious pancytopenia after CD7-CART which did not improve before allo-HSCT. On August 19th, 2021, the bone marrow was re-evaluated, and the results showed the leukemia was in CR with severe hypoplasia. On August 25th, 2021, the patient withdrew from the clinical trial and visited the transplantation department. At presentation, he had a high fever, slightly low blood pressure, hypoxemia, relieved by low-flow oxygen, and severe fatigue. Laboratory tests revealed severe pancytopenia, high C-reactive protein and procalcitonin levels, and negative blood cultures. Radiography revealed a lung infection, which improved with effective antibiotic treatment. In addition, CD7 CAR-T cells were detected in the peripheral blood, but CD7+ T lymphocytes were undetectable (**Supplementary Figure 3**). On August 30th, 2021, pre-HSCT conditioning was initiated with busulfan (0.8 mg/kg, every 6 h for 3 days), fludarabine (30 mg/m^2^, once daily for 5 days), and ATG (Fresenius Biotech GmbH, total dose 10 mg/kg). His father was also his HSCT donor. Haplo-identical donor-derived PBSCs containing MNC 24.54×10^8^/kg and CD34+ cells 3.94×10^6^/kg were infused on September 8th, 2021 (+35 days post-CAR-T). CsA, mycophenolate sodium, and a short course of MTX were used to prevent GVHD. CsA was administered from day -9, with adjustments to maintain a plasma concentration at 100–150 ng/ml in the first month post-HSCT. Mycophenolate sodium (6 mg/kg q12h) was administered from day -2 to day +28, and MTX was prescribed at doses of 15 mg/m^2^ on day +1, and 10 mg/m^2^ on days +3, +6, and +11. Tacrolimus was administered at a very low concentration for 11 months after CsA administration. Neutrophil cells and platelets recovered at +10 and +16 days post-HSCT, respectively. Complete donor chimerism of both CD3+ T cells and bone marrow-nucleated cells was achieved on day +30 post-HSCT, as analyzed by multiplex PCR using STRs.

No acute GVHD or infection occurred after transplantation. Six months post-HSCT, he showed lichenification in both arms, which improved with tacrolimus. All drugs were suspended at 12 months post-HSCT, and no events have occurred since then. We regularly reviewed the bone marrow, peripheral blood, and imaging tests to assess his condition (+1, +2, +3, +4, +6, +8, +10, +12, +15, +18, +21, and +24 months) following HSCT; leukemia-free survival (LFS) with MRD- detected by flow cytometry has been >2 years thus far. The CD7+, CD4+ and CD8+ cells recovery was shown in **Supplementary Figure 3**.

**Figure 3:**
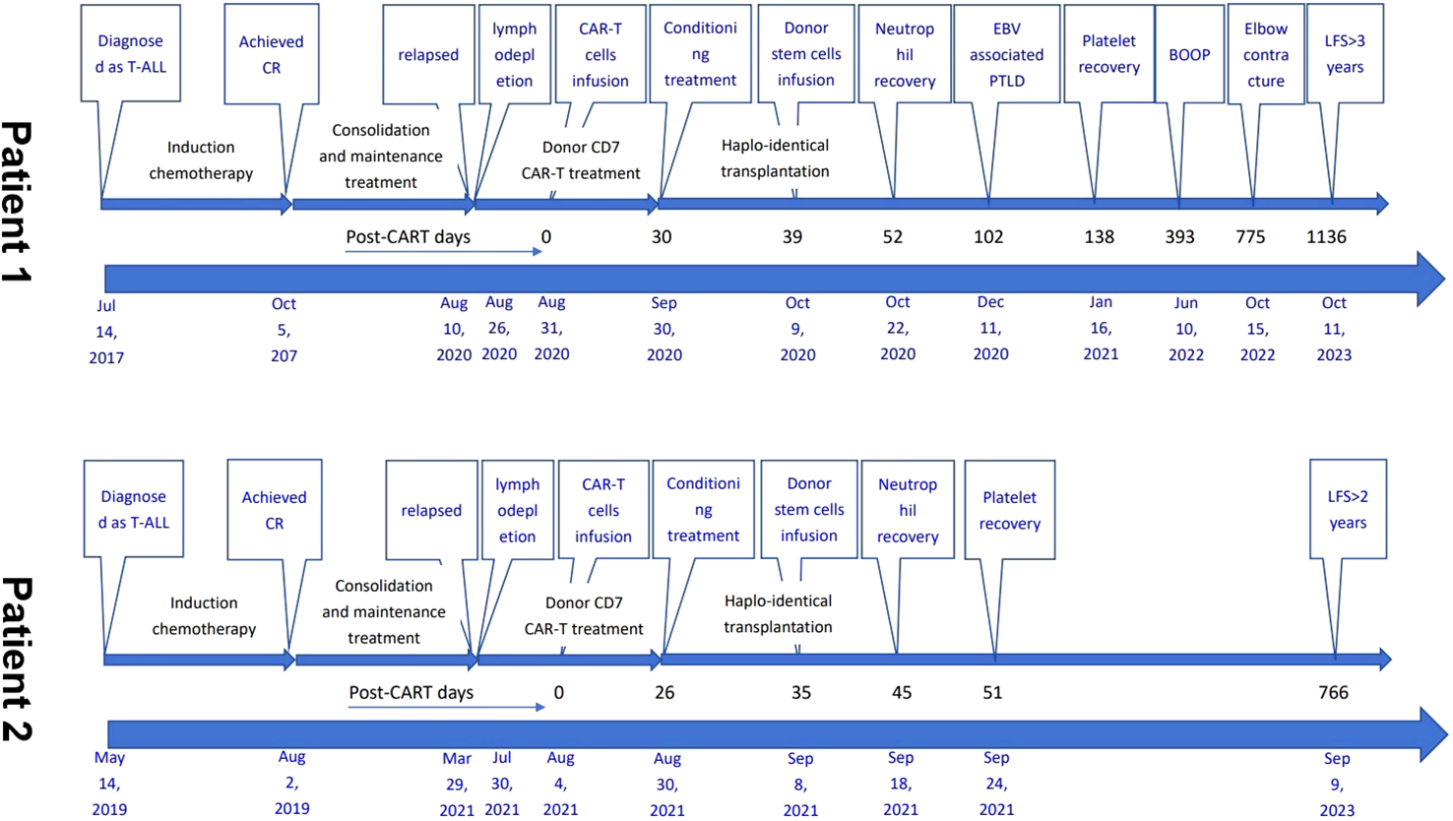
Timelines of the two patients’ disease course. T-ALL, acute T lymphoblastic leukemia; CR, complete remission; CAR-T, chimeric antigen receptor T; allo-, allogeneic; HSCT, hematopoietic stem cell transplantation; PBSCs, peripheral blood stem cells; EBV, Epstein-Barr virus; PTLD, post-transplant lymphoproliferative disease; BOOP, bronchiolitis obliterans with organizing pneumonia; LFS, leukemia-free survival.

The demographic and treatment details, and timelines of the disease course of the two patients are presented in **Table 1** and **Figure 3**.

**Table 1:** Demographic and treatment details of the two patients.

	Patient 1	Patient 2
Sex	male	male
Age (years)	10	8
Diagnosed date	July 2017	May 2019
Relapsed date	Aug 2020	March 2021
Personal history	No special	No special
Family history	No special	No special
Re-induction regimens	cyclophosphamide, idarubicin, vindesine, dexamethasone and L-asparaginase	mitoxantrone, dexamethasone and vincristine
lymphodepletion treatment beginning date before donor CD7 CAR-T	Aug 31^st^, 2020	Jul 30^th^, 2020
Conditioning regimens before CAR-T	Flu 30mg/m^2^ day1-3; cyclophosphamide 30mg/kg day1-3 (7)	Flu 30mg/m^2^ day1-3; cyclophosphamide 30mg/kg day1-3 (7)
Conditioning treatment beginning date before allo-HSCT	Sep 30^th^, 2020	Aug 30^th^, 2021
Conditioning regimens before allo-HSCT	Bu: 0.8mg/kg q6h from day −9 to day −7; Flu: 30mg/m^2^/d from day−6 to day−2; smolastine: 250mg/m^2^ day−3; ATG: total 15mg/kg	Bu: 0.8mg/kg q6h from day −9 to day −7; Flu: 30mg/m^2^/d from day−6 to day−2; smolastine: 250mg/m^2^ day−3; ATG: total 10mg/kg
Infusion date of donor stem cells	Oct 9^th^ and 10^th^, 2020	Sep 8^th^, 2021
Infused stem cells count (X10^6^/kg of recipient)	MNC: 5.1; CD34+cells: 4.4	MNC: 24.54; CD34+cells: 3.94
GVHD prophylaxis regimens	CsA, mycophenolate sodium and a short course of MTX	CsA, mycophenolate sodium and a short course of MTX
Neutrophil cells engraftment date	+12 days post-HSCT	+10 days post-HSCT
Platelets engraftment date	3 months post-HSCT	+16 days post-HSCT

CAR-T, chimeric antigen receptor T cells; Flu, fludarabine; allo-, allogeneic; HSCT, hematopoietic stem cell transplantation; Bu, busulfan; ATG, anti-human T-cell lymphocyte rabbit immunoglobulin (Fresenius Biotech GmbH); MNC, mononuclear cells; GVHD, graft-versus-host-disease; CsA, cyclosporine; MTX, methotrexate.

## 3 Discussion

In recent years, the progress in modern chemotherapy regimens has improved the prognosis of T-ALL (13–15). However, when the disease relapses or is refractory to induction therapy, most patients have a very poor prognosis and can no longer be cured (16). In 2005, Nelarabine was approved for r/r T-ALL treatment and achieved CR in 30–40% of patients (17, 18). In addition to nelarabine, liposomal vincristine and daratumumab can also be used to treat r/r T-ALL; however, both resulted in a small proportion of patients in remission (19–21). These new agents do not achieve CR in most patients. To significantly improve the prognosis of r/r T-ALL, researchers have attempted to develop CAR-T for T-ALL. There is no unique target on T-ALL cells that is not present on healthy T cells and the “fratricide” that the engineered T cells will target each other is the first problem to resolve (22). In 2017, Yi Tian Png et al. reported a novel approach to block CD7 expression in T cells (23). In 2018, Cooper et al. reported that “off-the-shelf” fratricide-resistant CD7 CAR-T cells were effective in treating patient-derived xenograft T-ALL mice (24). In 2021, doctor Pan in our hospital published the first phase I trial of donor-derived CD7 CAR-T cells for r/r T-ALL, which showed promising results with 90% of patients achieving CR (7). For patients who had not received HSCT before CAR-T, long-term pancytopenia and CD7+ T-cell aplasia could occur; therefore, these patients should be bridged to allo-HSCT. Li et al. has shown the effectiveness and feasibility of bridging donor CD7 CAR-T cells to allo-HSCT (25). In these two cases, we chose CD7 as the CAR-T target and donor-derived anti-CD7 CAR-T cells to avoid contamination with malignant T cells. Our CAR-T cells showed rapid expansion and complete elimination of leukemia cells. In the two patients, when CAR-T cells were infused, they increased exponentially, accompanied by the rapid disappearance of CD7+ cells in the peripheral blood, and effectively put leukemia into CR. These results suggest that donor CD7 CAR-T cells are an effective treatment to salvage r/r T-ALL.

As described by Pan et al., patients who receive donor CD7 CAR-T cells and have not received HSCT will develop persistent pancytopenia (7). Following CAR-T cell infusion, the two patients developed severe pancytopenia with grade 2 CRS. After CRS, the white blood cells partially recovered, but decreased again in <10 days, which was only improved by allo-HSCT (**Figure 2**). We considered that CRS accounted for the quickly occurring pancytopenia, while GVHD caused by the allograft of CAR-T cells suppressed blood cell production, which was the reason for the subsequent reduction in WBCs. This process is similar to transfusion-associated GVHD (26). Most CAR-T cells are eliminated in the preparative treatment before allo-HSCT, and donor hematopoietic stem cells are infused during transplantation for hematopoiesis recovery after allo-HSCT (**Figure 2**). Doctor Li reported that 12 patients received allo-HSCT following infusion with donor CD7 CAR-T; donor stem cells in all patients except for one successfully engrafted with complete donor chimerism and blood cell recovery (25). Patient 1 developed a severe virus infection after CAR-T until three months post-HSCT which caused delayed engraftment of platelets. After controlling EBV-associated PTLD, his platelet counts gradually recovered to normal levels. In Patient 2, the neutrophils and platelets recovered at +10 and +16 days post-HSCT, respectively, similar to other patients who received haploidentical HSCT.

CD7 molecules are commonly expressed on the surface of normal T cells, natural killer cells, and malignant T cells (27, 28). Following CD7 CAR-T cell infusion, both normal and malignant CD7+ cells are eliminated; therefore, patients who receive treatment have a high risk of infection (7). Both patients had serious infections after CAR-T cell infusion; Patient 1 had viral hemorrhagic cystitis and HHV6 viremia, and Patient 2 had lung infection before transplantation. After HSCT, Patient 1 developed EBV-associated PTLD and CMV retinitis. Patients receiving donor CD7 CAR-T cells bridging allo-HSCT are also at a high risk of infection in the early stage post-HSCT because of CD7+ T cell aplasia caused by the residual CD7 CAR-T cells after transplantation (25). When CD7 CAR-T cells disappeared and CD7+ T lymphocytes recovered, the risk of infection decreased (**Supplementary Figure 3**). Patient 2 did not develop infection post-HSCT, indicating that not all patients will develop serious infection post-HSCT. To reduce the risk of infection, we reduced the ATG dose during the transplantation process which did not influence the engraftment of donor stem cells.

After CD7 CAR-T treatment, infection risk increases before recovery of CD7+ T cells post-HSCT. Effective prophylaxis and anti-infection treatment should be prescribed to reduce the infection risk.

As mentioned above, patients who receive donor CD7 CAR-T cells can develop GVHD before transplantation (7); both patients presented with GVHD-like symptoms after CAR-T cell infusion before allo-HSCT. However, for patients who received donor CD7 CAR-T cells bridging allo-HSCT, the post-HSCT GVHD risk was not significantly different from that of other patients receiving allo-HSCT (25). Patient 1 had acute GVHD and extensive chronic GVHD, whereas Patient 2 only had limited chronic GVHD. The reduction in ATG dose also controlled the risk of GVHD.

In 2022, Hu et al. (29) and Lu et al. (30). respectively reported genetically modified CD7 CAR-T for CD7-positive hematological malignancies and naturally selected CD7 CART for T-ALL, in which 3 and 14 patients were bridged to allo-HSCT; and all patients have been in CR for more than 10 and 6 months. In 2023, Chiesa et al. (31) reported three T-ALL patients who received base-edited CD7 CAR-T, of which two achieved CR and were bridged to allo-HSCT (31). In the two reported cases, CD7 CAR-T eliminated leukemia cells, and the two patients achieved CR and MRD- before allo-HSCT which significantly reduced the relapse risk compared with salvage allo-HSCT. Allo-HSCT consolidated the remission status by graft versus leukemia effect and restored their immune function post CAR-T. CD7 CAR-T cell therapy as a bridge to allo-HSCT induce long term remission in patients with r/r T-ALL, otherwise uncurable. With a follow up > 2 years, the patients are in remission and resume a normal life. Therefore, our findings suggest that donor CD7 CAR-T bridged to allo-HSCT may cure r/r T-ALL, similar to r/r B-ALL.

## 4 Patient perspective

After receiving donor CD7 CAR-T and HSCT, the two patients achieved CR and have led healthy lives for >2 years. In the past, patients with relapsed T-ALL had poor prognosis but there is a possibility of cure. We believe that relapsed T-ALL can now be considered a curable disease with this treatment.

## Data availability statement

The raw data supporting the conclusions of this article will be made available by the authors, without undue reservation.

## Ethics statement

Written informed consent was obtained from the minor(s)’ legal guardian/next of kin for the publication of any potentially identifiable images or data included in this article.

## Author contributions

YS: Data curation, Investigation, Writing – original draft, Writing – review & editing. ZL: Writing – original draft. QW: Writing – original draft. KG: Writing – original draft. TW: Validation, Writing – original draft, Writing – review & editing.
