# Supplementary figures and images for: Case report: Two pediatric cases of long-term leukemia-free survival with relapsed acute T-lymphoblastic leukemia treated with donor CD7 CAR-T cells bridging to haploidentical stem cell transplantation
Source: Front Immunol. 2024 Feb 28;15:1333037. doi: 10.3389/fimmu.2024.1333037 (PMC10934222; doi:10.3389/fimmu.2024.1333037)

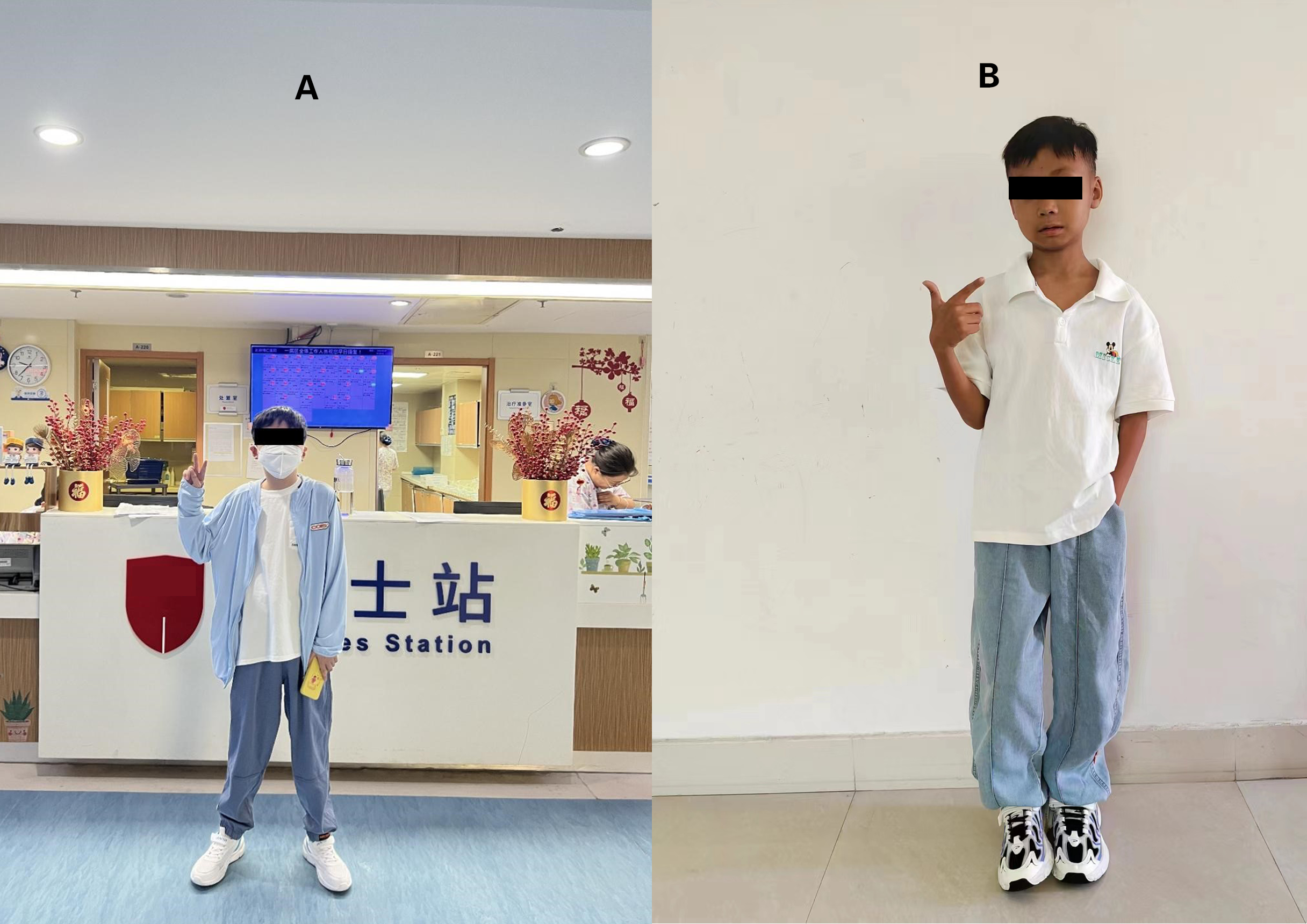

Supplement: Supplementary file 1 [file Image_1.png]

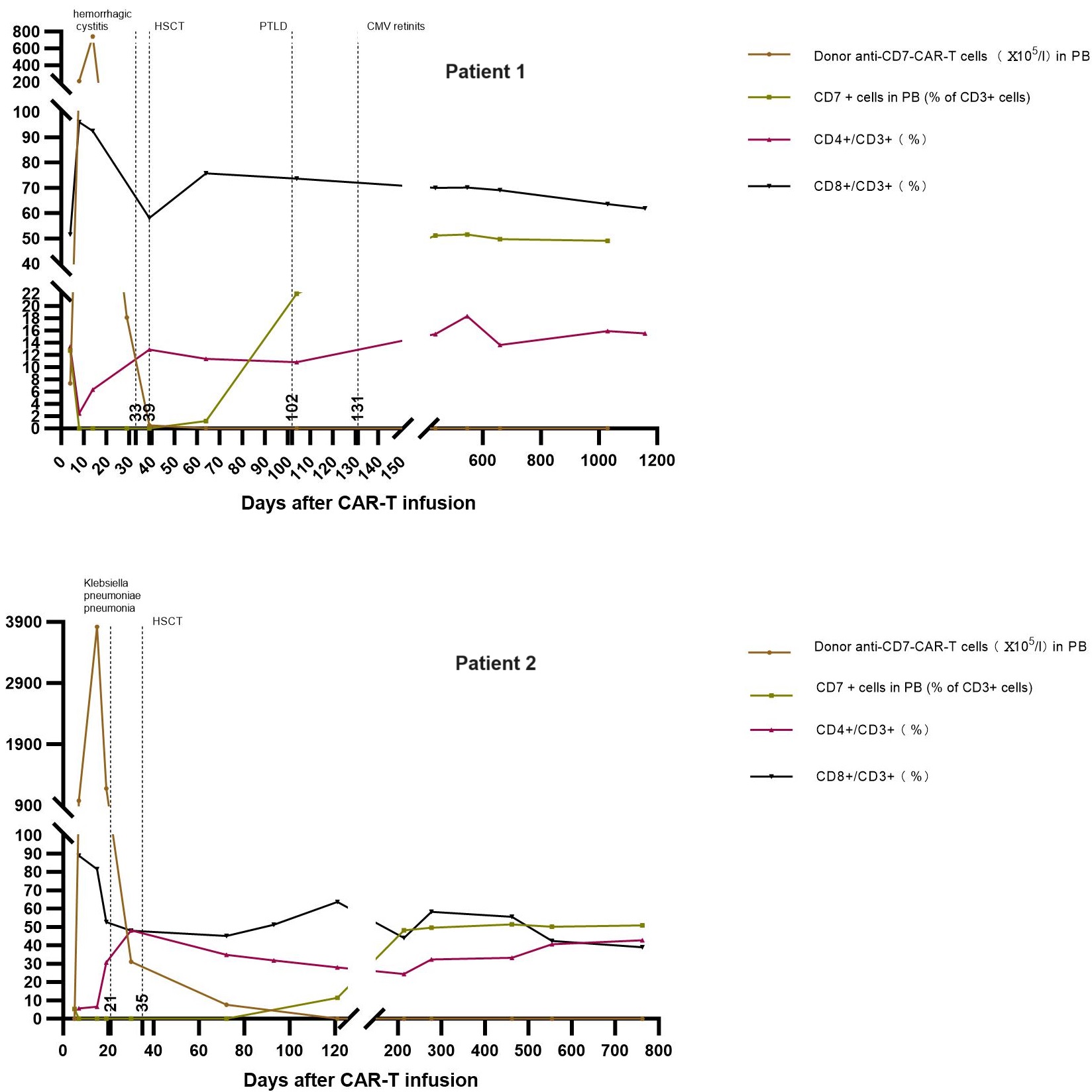

Supplement: Supplementary file 2 [file Image_2.jpeg]

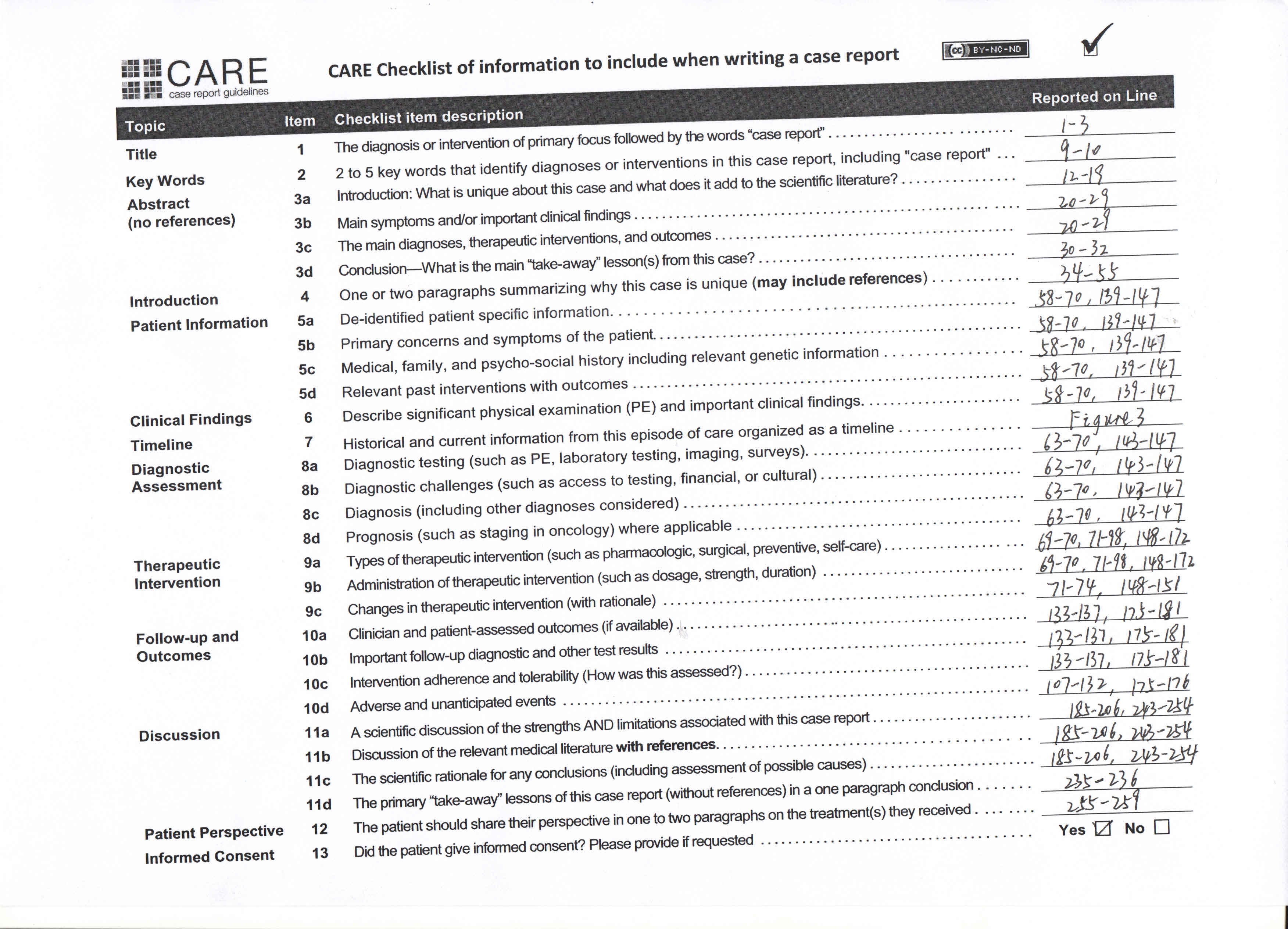

Supplement: Supplementary file 3 [file Image_3.jpeg]
